# Calcaneal Plate for Medial Femoral Condyle Fractures—Is This It? A Clinical Case

**DOI:** 10.1055/s-0041-1725159

**Published:** 2021-05-25

**Authors:** Rómulo Silva, Elsa Moreira, Ricardo Branco, Filomena Ferreira, Margarida Areias, Carolina Oliveira, Bruno Alpoim

**Affiliations:** 1 Department of Orthopedics and Traumatology, ULSAM – Viana do Castelo, Portugal

**Keywords:** knee, trauma, calcaneal, plate

## Abstract

Management of unicondylar femoral fractures is mainly done by open reduction and internal fixation. Anatomic reduction in the articular surface is paramount in this type of lesion. Medial condyle fractures lack specific osteosynthesis material for fixation.

We report a case resolved with the sparsely documented technique using calcaneal plate fixation.

Unicondylar fractures of the distal femur represent less than 1% of all femoral fractures. 1 2 This type of lesion is frequently associated with other injuries of the limb. 3 4 5 These often occur after avulsion, direct impact or shear force on the knee, mostly during sports or dashboard trauma in the setting of traffic accidents. 2 5

There is a strong recommendation in literature for open reduction and internal fixation of this type of injuries. 3 4 5 6 Many fixation implants and techniques have been advocated to treat these injuries, from cannulated screws to conventional and locking plates. 7 The majority of materials are precontoured to, the most frequently injured, lateral condyle, with the medial condyle being usually addressed indirectly. 3 4 5 Reports of applicability of a calcaneus plate for fixation of the medial condyle are rising. 3 4 5

The patient provided written informed consent for print and electronic publication of this case report.

## Case Report

**Fig. 1:**
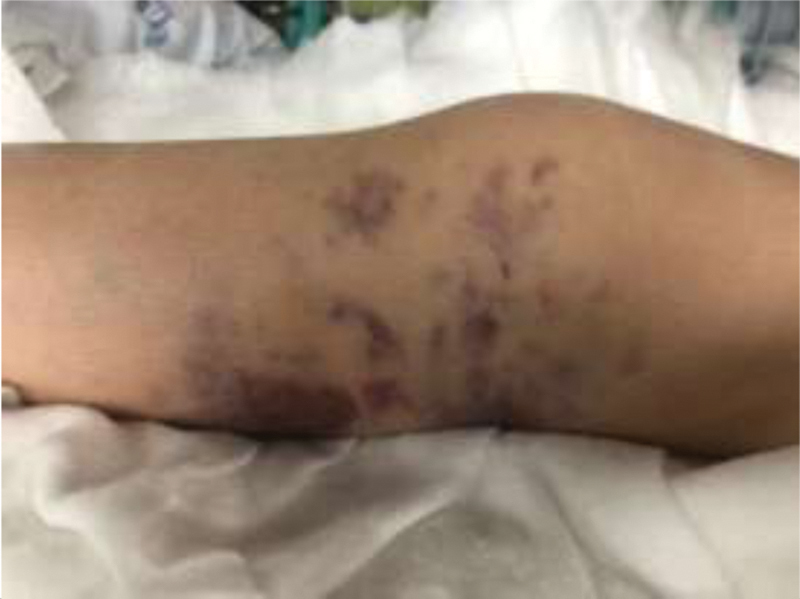
Effusion and ecchymosis of the injured knee.

Our patient is a 16-year-old female who was a victim in a car crash. She had no relevant medical or familiar background. She complained of isolated knee pain, incapacity to bear weight, and difficult range of motion. On physical examination, there were no apparent neurovascular injuries of the limb. The knee had significant effusion and a lateral ecchymosis ( Fig. 1 ). The patient also showed varus instability upon stress testing.

**Fig. 2:**
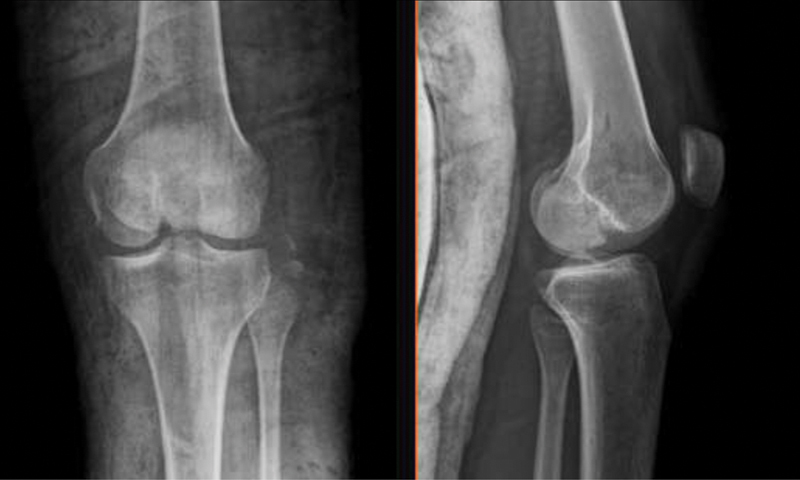
Anteroposterior and lateral view of radiographs.

**Fig. 3:**
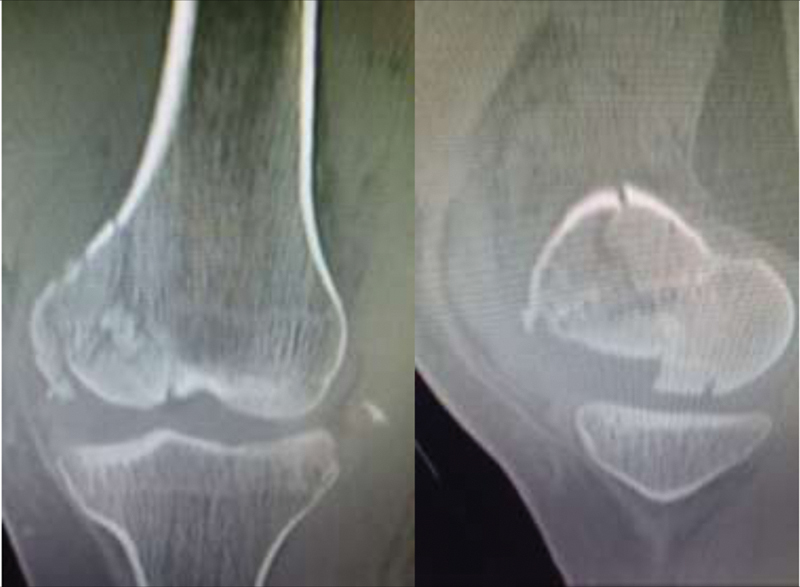
Computed tomography scan—coronal and sagittal cuts.

**Fig. 4:**
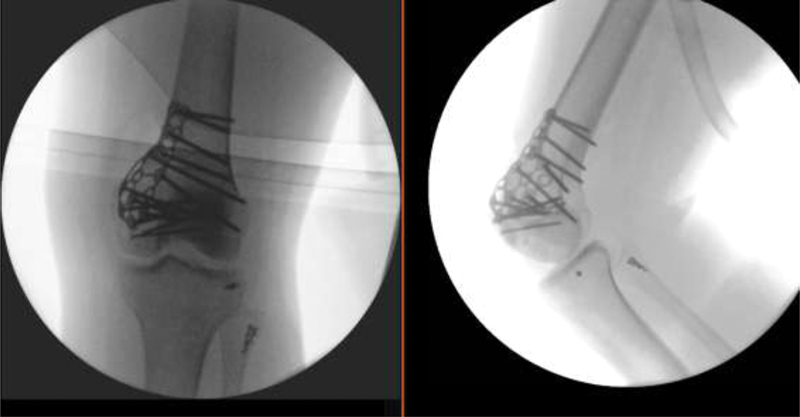
Anteroposterior and lateral view intraoperative radiographs.

Radiographs and CT scan showed a comminuted femoral medial condyle fracture associated with avulsion of the lateral collateral ligament from the fibular head ( Figs. 2 and 3 ). She was submitted to open reduction via anteromedial incision with a subvastus approach and fixated with a D-shaped calcaneal plate associated with two isolated total threaded cancellous screws and autologous graft from the iliac crest. Lateral collateral ligament and iliotibial band were reattached with anchors, after fibular nerve exploration ( Fig. 4 ).

**Fig. 5:**
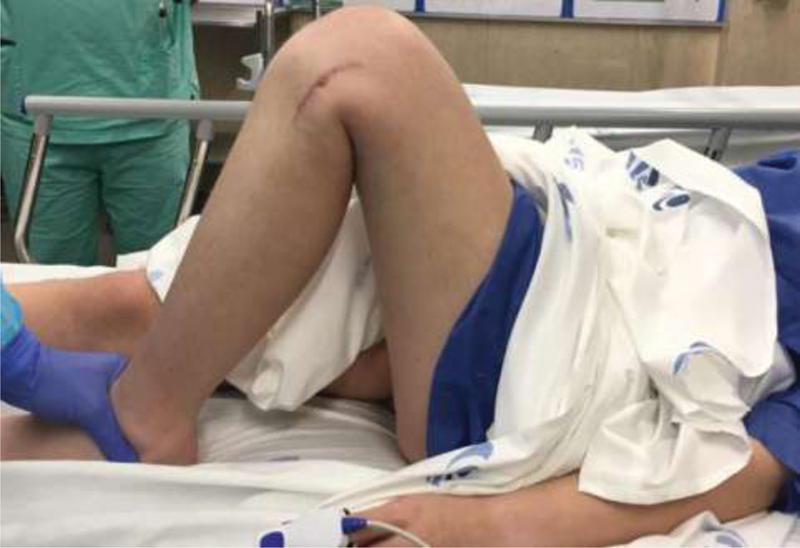
Six months knee range of motion.

At 6-month follow-up, the patient had a knee range of motion from 0 to 110 degrees and weight-bearing as tolerated ( Fig. 5 ).

## Discussion

The treatment options for distal femoral fractures range from conservative to open reduction and internal fixation. 5 In displaced fractures, the surgical treatment is consensual. 3 4 6 7 The purpose of surgical treatment is to restore anatomic articular surface and maintain alignment, length, and rotation. 3 4 5 6 To prevent stiffness and ease recovery, early range of motion is paramount, which in turn depends on stable fixation. 3 4

Since the medial femoral condyle lacks specific implant designs, there are growing reports using calcaneal plates for these injuries. 3 4 5 Calcaneal plate has several features that make it appropriate 3 4 5 : Large area of bone coverage, which increases the fixation surface; spanning structural design with possibility of intermittent fixation; various options for screw placement with adaptability to different fracture patterns and bone quality; low profile design, reducing the risk of conflict with surrounding tissues; malleability with maintained durability and strength. These characteristics allow this plate to be a serious management option for osteopenic, osteoporotic bone, and comminuted fractures. 3 5 The applicability and good results of this technique have been reported in literature. Hohman et al described, in 2012, two cases using calcaneal plates for unicondylar fractures, one lateral and one medial sided, with promising results. 3 In 2015, Loesch et al reported another case of medial femoral condyle fracture treated with calcaneal plating. 4 An observational study with 17 patients from Meena et al showed excellent function outcomes with this method of fixation. 5 All the accounted cases are of unicondylar fractures that underline the inapplicability of this construct for bicondylar patterns, as they lack metaphyseal stability. 3 4 5

## Conclusion

Promising results are present in literature regarding the management of unicondylar fractures with calcaneal plates. A large randomized control trial is required to support these good results and possibly standardize this technique for medial condyle displaced fractures.
